# Preference evaluation of ground beef by untrained subjects with three levels of finely textured beef

**DOI:** 10.1371/journal.pone.0190680

**Published:** 2018-01-17

**Authors:** Sandra Molly Depue, Morgan Marie Neilson, Jayson L. Lusk, Gretchen Mafi, F. Bailey Norwood, Ranjith Ramanathan, Deborah VanOverbeke

**Affiliations:** 1 Department of Agricultural Economics, Oklahoma State University, Stillwater, Oklahoma, United States of America; 2 Department of Animal Science, Oklahoma State University, Stillwater, Oklahoma, United States of America; University of Florida, UNITED STATES

## Abstract

After receiving bad publicity in 2012 and being removed from many ground beef products, finely textured beef (referred to as ‘pink slime’ by some) is making a comeback. Some of its proponents argue that consumers prefer ground beef containing finely textured beef, but no objective scientific party has tested this claim—that is the purpose of the present study. Over 200 untrained subjects participated in a sensory analysis in which they tasted one ground beef sample with no finely textured beef, another with 15% finely textured beef (by weight), and another with more than 15%. Beef with 15% finely textured beef has an improved juiciness (p < 0.01) and tenderness (p < 0.01) quality. However, subjects rate the flavor-liking and overall likeability the same regardless of the finely textured beef content. Moreover, when the three beef types are consumed as part of a slider (small hamburger), subjects are indifferent to the level of finely textured beef.

## Introduction

Meat processors have reduced the labor needed to convert livestock to meat by about 80% since 1970, leading to higher prices for livestock producers and lower prices for consumers [1]. Two recent innovations are advanced meat recovery and lean, finely textured beef. Both allow the capture of beef for human consumption that would otherwise be used for lower-value products like pet food—but the latter is far more controversial than the former. Advanced meat recovery simply uses machinery to separate beef from the bone in ways a human cannot [2], while lean, finely textured beef requires heat and often a microorganism inhibitor like ammonium hydroxide or citric acid.

Beef ensconced in fat was once too difficult to separate for human consumption, but innovations begun in the 1980s and refined in the last twenty years have allowed processors to retrieve much of this beef and include it in the ground beef supply. The lean, finely textured beef process works by first heating the beef/fat trimmings to about 42°C and then spinning it in a centrifuge to separate the meat from the fat. Because heat encourages microorganism growth, the beef is treated with ammonium hydroxide before it is pressed and frozen. The resulting meat is simply beef, though leaner and more finely textured, and is thus referred to as lean, finely textured beef, or just finely textured beef (FTB) [3,4]. The process has been extensively studied, is approved for use by the United States Department of Agriculture (USDA), and is considered by the Food and Drug Administration to be in a category of “Generally Recognized as Safe” (GRAS).

Meat scientists and regulators may approve of the FTB process, but between 2009 and 2012 the American public developed a different opinion [5,6]. Readers who have not heard the term ‘finely textured beef’ are likely to be familiar with its moniker: pink slime. The moniker’s first recorded use was in an internal USDA memo from scientist Gerald Zirnstein. It refers to the FTB’s different color and texture compared to other beef cuts. The moniker was made public in a 2009 *New York Times* article by Michael Moss, who questioned its safety. Two years later, Jamie Oliver devoted a whole episode of *Food Revolution* to chastising FTB, and when ABC News ran an equally critical story, the future of FTB seemed bleak. A number of restaurants and grocery stores announced it would no longer carry beef products containing FTB. The major FTB producer closed three of its four plants and cut more than 700 jobs. Other FTB producers did likewise. Yet, during this time there was little to no opposition to FTB by food scientists or government officials, probably because it had been studied for decades and no evidence had emerged of its dangers [5,6].

The overall impact of the pink slime controversy, however, was slight and short-lived [6,7], and as higher beef prices made efficient harvesting ever more important, this shunned beef product began returning to the market. Relative to its low point in 2012, production of FTB has doubled [8], with little apparent concern by the media or the public. With the return of FTB, and no apparent safety concerns being debated in the public forum, it is worth asking whether there are other benefits of FTB besides lowering the cost of ground beef production. Private sources have claimed that adding FTB to ground beef improves its tenderness, texture, and taste [9,10], but no objective scientific investigation of this claim (by scientists who are not paid by the companies making the product) has been pursued—that is the purpose of the present study.

There are reasons to believe the taste of ground beef might change with the addition of finely textured beef. Its addition increases the reducing capacity (ability of beef to limit metmyoglobin formation/meat discoloration) of the beef, perhaps due to the release of pro-oxidants from membrane bound organelle during freezing and thawing of FTB. It can increase the pH of meat as well as its mineral content, and it makes the meat more susceptible to premature browning. Its color differences before cooking include a less dark (higher L* value), redder (higher a* value), and less yellow (lower b* value) color, but after cooking those differences disappear [3,4].

Given that FTB is a public issue, an evaluation by independent scientists to appraise its sensory merits is warranted. This study uses sensory instruments similar to other studies. Following Zimoch and Gullett [11], the beef is evaluated according to its juiciness and tenderness, and like Morales *et al*. [12], flavor is included in addition to overall satisfaction. These attributes are rated by subjects using the familiar 9-Point Hedonic Scale [13], where a higher number indicates greater likeability. In the spirit of Napolitano [14], after the beef is studied using the hedonic scales, the subjects are asked to indicate their willingness-to-pay for the products. Instead of using Vickrey auctions, though, a series of hypothetical choice experiments similar to those used by both Lagerkvist [15] and Dominguez-Torreiro [16] are conducted.

## Material and methods

**Fig 1:**
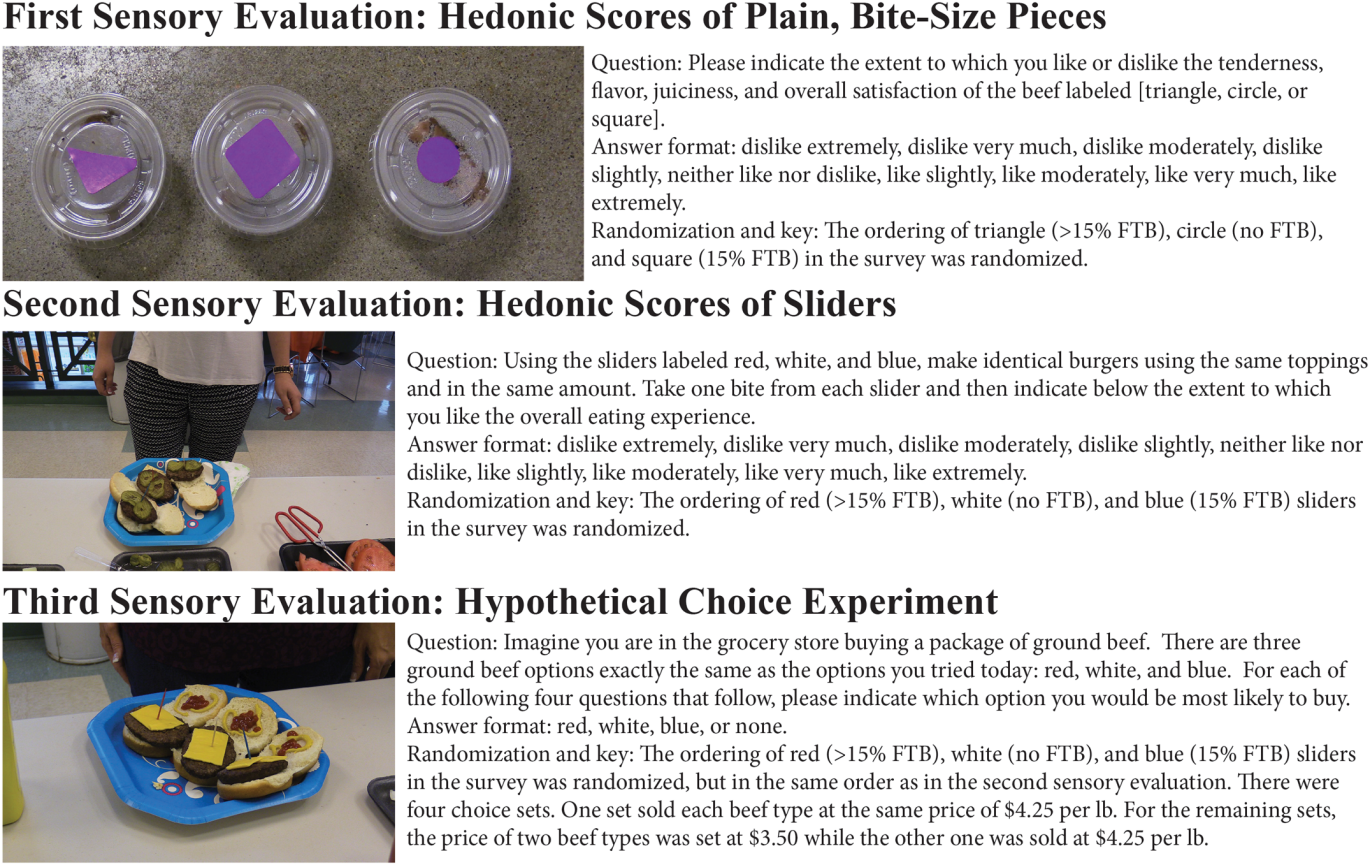
Illustration of three sensory evaluations.

Untrained subjects are asked to taste ground beef made with three different levels of finely textured beef, without any information other than it is ground beef prepared by a meat scientist and is as safe as a normal meal. Untrained, as opposed to trained, subjects are used because the research objective is to test whether regular consumers can discern between beef with varying levels of finely textured beef (FTB) [17]. At no point are the subjects informed about the use of FTB, nor is the term ‘pink slime’ ever used. The research thus measures the impact of FTB on the taste of ground beef, independent of people’s perceptions of the process or reactions to terms like ‘pink slime.’ Images of the experiment and the questionnaire used are provided in Fig 1, where the reader can see that the beef is identified by shape or color, not according to its level of FTB.

### Food samples

To isolate the influence of finely textured beef (FTB) on taste in ground beef, identical ground beef patties are acquired from a meat processor and used in a sensory analysis. Each patty is the size of a slider (2.5 inches diameter, 0.5 inches thick before cooking). All are 81% lean and 19% fat (with a variance of +/- 1%) but contain either 0% FTB, 15% FTB, or more than 15% FTB, by weight. The exact level of FTB in the > 15% FTB category is proprietary. These products are obtained from a modern beef processing plant using the same equipment, personnel, and methods used to process FTB for consumers.

The patties arrive frozen and in three different packages, each containing one of the three ground beef types. The packages are not labeled by their FTB content but by a generic number. It is not until after the study is complete and the data are analyzed that the researchers are informed of the level of FTB in each type of beef, thereby making the research a double-blind design.

For cooking, the patties are thawed at 4°C for 24 hours and then cooked in an XLT Impingement Oven (Model 3240-TS, BOFI Inc., Wichita, KS) at 204°C, reaching an internal temperature of 74°C (using Atkins, AquaTuff 351 thermometer). This internal temperature is slightly higher than USDA requirements and is chosen to make absolutely sure the minimum USDA temperature is reached. Cooking is performed immediately prior to the sensory analysis, and all patties are placed in a warmer between the time they are finished cooking and the time they are consumed. Approximately 20 minutes pass between the time the patties are finished cooking and until they are consumed as sliders.

### Subjects

Two types of individuals are recruited for the sensory analysis. One group consists of undergraduate students residing in a common dormitory at a major U.S. university in the Midwest, and they are compensated $10 in cash for their participation. They are recruited directly at meetings and through signs posted in their dormitory. The second group consists of faculty and staff at the same university, recruited using word of mouth and email invitations; each is given a $10 Walmart gift certificate for participating. In the recruiting materials the subjects are told, “During the research project, you will indicate your like/dislike for tenderness, juiciness, and flavor profiles of ground beef samples and will be asked to compare beef sliders and to indicate your purchasing preferences.” No other information about the food is provided to them. During the experiment sessions they are only told of the meat that, “All food has been prepared by a meat scientist and so will be as safe as a normal meal.” No mention of the differences in the ground beef types is provided.

A total of 232 subjects participate, 60% being students and 40% being non-student adults. There are more females (62%) than males (38%), as shown in Table 1. All are familiar with beef, with 85% reporting that they consume ground beef frequently and 15% consuming it rarely. No subjects indicate that they never consume beef. Four different research sessions are held for the students and a separate four sessions are held for the adults, all in the fall of 2014; each participant attends only one session. After removing responses from individuals who did not complete all of the hedonic score questions, a total of 222 subjects remain. This research design was approved by the Oklahoma State University Institutional Review Board (application number AG1441).

**Table 1:** Demographics of the 222 subjects.

Demographic	Descriptive Statistic
***Gender***	
Male Female	38% 62%
**Age**	
Mean Standard Deviation	30 14
**Frequency of burger consumption**	
Frequently Rarely Never	76% 23% 1%
**Frequency of ground beef consumption**	
Frequently Rarely Never	85% 15% 0%
**Frequency of ground beef purchases**	
Once a week	36%
Once every two weeks	30%
Once a month	14%
Once every two months	5%
Less than once every two months	8%
Never	7%
**Household Income Before Taxes**	
Less than or equal to $50,000 More than $50,000	36% 64%

### First sensory evaluation: Plain beef samples

All evaluations are completed in a classroom at the university’s food processing center. Though the university has a standard ISO sensory room, more space is needed to accommodate the larger groups of subjects. Extra space is also needed for subjects to be able to go through a buffet line and construct their own sliders (as described in the next section). Moreover, as will be demonstrated in the second sensory evaluation, part of the experiment’s objective is to better mimic a realistic eating experience, so subjects are able to converse with one another at one part of the experiment (as they would in a normal meal), and booths in a sensory room would make this difficult. Note that the classroom has plenty of space for the subjects and is rather plain, with no particular colors or signs that might influence preferences, and there is a long precedent for studies performing sensory analysis—especially of meats—outside of standard sensory rooms [18, 19, 20, 21, 22, 23, 24, 25].

The first evaluation is a blind taste test using bite-size pieces of ground beef cooked without any flavorings added. Each subject is given a tray containing three containers, each identified by the shape of a square, triangle, or circle, as shown in Fig 1. Each shape corresponds to one of the three beef types (no FTB, 15% FTB, > 15% FTB), but again, the researchers are unaware of the FTB content associated with any shape until the data analysis is complete. Only after the completion of the initial manuscript was it revealed that (1) ‘circle’ is the no FTB sample (2) ‘square’ is the 15% FTB sample, and (3) ‘triangle’ is the sample with more than 15% FTB.

Along with the beef are unsalted crackers, toothpicks, and bottles of water. Subjects are given a questionnaire directing them to always take a bite of the cracker and a drink of water before tasting each sample. They are then given directions on which shape to taste first, and this part is randomized such that approximately one-third tastes the square sample first, one-third tastes the circle first, and one-third tastes the triangle first. There are three different orderings of shapes used: (1) square, triangle, then circle, referred to as Order A; (2) triangle, circle, then square (Order B); and (3) circle, square, then triangle (Order C). In the experiment 30% of the subjects face Order A, 38% face Order B, and 32% face Order C. Talking among the subjects is prohibited for this part of the experiment, and no information is given about the beef other than it was processed and cooked in a regulated facility under the supervision of meat scientists.

After taking each bite, the subject is asked to rate the tenderness using the 9-Point Hedonic Scale: 9 = like extremely / 8 = like very much / 7 = like moderately / 6 = like slightly / 5 = neither like nor dislike / 4 = dislike slightly / 3 = dislike moderately / 2 = dislike very much / 1 = dislike extremely. The same rating system is used for flavor, juiciness, and satisfaction with overall eating quality. Using this conventional hedonic scale, subjects can enter the same score for any two beef products if they wish. The full questionnaire is provided in S1 Appendix. There, one can see that subjects are asked to score each beef type separately, but there is nothing preventing subjects from going back and changing their answers if they like.

The hedonic scores are first evaluated by comparing the averages for the three different beef types in a visual representation of the raw data results. Also, factor analysis is used to identify any latent constructs helping to explain variations in the attribute ratings. Hypothesis tests are not performed on the raw data because simple tests like ANOVA do not account for the ordinal, discrete nature of the hedonic scores. Moreover, it is preferable to account for demographics and the order in which the three beef samples are tasted when testing for differences [26]. Due to the nature of the hedonic scores, an ordinal-logit model is employed, as specified in (1).

This model assumes the latent (unobserved to the researcher but not the subject) score for any one attribute—tenderness, juiciness, flavor, or overall satisfaction—is generated by the following equation. The overall level of the latent scores are influenced by the order in which the samples are tasted, gender, age, the frequency the subject eats burgers, and household income. The statistical significance of these variables is not evaluated, as a later section employees a tool for multiple testing, and the more tests conducted the weaker the p-value from any individual test (only p-values relevant to the study’s objectives are evaluated) [27]. Thus, the explanatory variables are used to hold constant any effects that might exist, not to test for their existence.

The frequency of burger consumption is used instead of ground beef consumption or ground beef purchases because the second evaluation considers burgers specifically and the beef samples are presented not as crumbled beef but in patty form.

$$\begin{array}{l} U_{ik}=V_{ik}\;+\;\tau_{i}+\epsilon_{ik}\\ =\left\{\beta_{1}\left(noFTB_{ik}\right)+\beta_{2}\left(15FTB_{ik}\right)+\beta_{3}\left(maxFTB_{ik}\right)\right\}\\ +\{\alpha_{1}\left(orderA_{ik}\right)+\alpha_{2}\left(orderB_{ik}\right)+\alpha_{3}\left(orderC_{ik}\right)+\alpha_{4}\left(female_{ik}\right)\\ +\alpha_{5}\left(age_{ik}\right)+\alpha_{6}\left(eatburgers_{ik}\right)+\alpha_{7}\left(income50_{ik}\right)\}+\tau_{i}+\epsilon_{ik} \end{array}$$ (1)

In (1), the dependent variable, *U*_*ik*_, represents the latent score subject *i* would assign to sample *k* if they could do so on a continuous scale. The first three explanatory variables, *noFTB*, *15FTB*, and *maxFTB*, are indicator variables denoting whether the sample contains no FTB, 15% FTB, or greater than 15% FTB. The variables *orderA*, *orderB*, and *orderC* are indicator variables denoting the three possible orders in which the beef samples are consumed (as described in the previous section). The variables *female*, *eatburgers*, and *income50* are indicator variables for females, those who consume burgers frequently (as opposed to rarely or never), and those with household incomes greater than $50,000, respectively. *Age* is the age of the subject. The subscript *i* = 1, …, 222 denotes the subject and the subscript *k* = noFTB, 15FTB, and maxFTB denotes the beef sample. Because each subject will consume each of the three beef samples, there is an individual-level stochastic term, τ_i_, assumed to follow a normal distribution with a zero mean and a standard deviation (σ) that must be estimated, making (1) a random-effects model, accounting for the fact that any one individual evaluates multiple samples. Also, there is a stochastic term unique to each subject-sample combination, denoted ε_ik_, assumed to follow the logistic distribution (with a zero mean and a scale parameter of 1).

Although the true appraisal of tenderness, juiciness, flavor, and overall satisfaction for any one individual is assumed to be a continuous function, only discrete values concerning their scores can be observed. That is, the exact value of *U*_*ik*_ is unobserved, but information is available on its lower- and upper-bound. So, rather than observing the actual latent score for subject *i* and sample *k*, we only observe the hedonic score *Y*_*ik*_, marked on the questionnaire, which is assumed to follow the function below, where the η_j_ are parameters to be estimated simultaneously with the α _j_, β _j_, and σ.

$$\begin{array}{l} Y_{ik}=1=dislike\;extremely\;if\;U_{ik}\leq \eta_{1}\\ Y_{ik}=2=dislike\;very\;much\;if\;\eta_{1}<U_{ik}\leq \eta_{2}\\ Y_{ik}=3=dislike\;moderately\;if\;\eta_{2}<U_{ik}\leq \eta_{3}\\ Y_{ik}=4=dislike\;slightly\;if\;\eta_{3}<U_{ik}\leq \eta_{4}\\ Y_{ik}=5=neither\;like\;nor\;dislike\;if\;\eta_{4}<U_{ik}\leq \eta_{5}\\ Y_{ik}=6=like\;slightly\;if\;\eta_{5}<U_{ik}\leq \eta_{6}\\ Y_{ik}=7=like\;moderately\;if\;\eta_{6}<U_{ik}\leq \eta_{7}\\ Y_{ik}=8=like\;very\;much\;if\;\eta_{7}<U_{ik}\leq \eta_{8}\\ Y_{ik}=9=like\;extremely\;if\;\eta_{8}<U_{ik} \end{array}$$ (2)

The parameters α _j_, β _j_, η_j_, and σ are estimated using maximum likelihood of the random effects ordered logit model using STATA (xtologit command). However, the parameters β_1_ and α_1_ are normalized to equal zero, and the value of all explanatory variables are set to zero whenever the beef sample contains no FTB, for model identification. This essentially normalizes the utility of beef without FTB to equal zero.

Separate models are estimated for the tenderness, juiciness, flavor, and overall satisfaction attributes. Each model is used to determine whether subjects assign different tenderness, juiciness, flavor, and overall satisfaction scores for the different beef types. If they do prefer the qualities of beef with a different level of FTB, then at least two of coefficients from β_1_, β_2_, and β_3_ will differ. Thus, to test whether varying levels of FTB alter its sensory properties, one can test the null hypothesis that the coefficients for all three beef types equal zero: β_1_ = β_2_ = β_3_ = 0, which would be true if subjects perceive the hedonic score for the attribute under consideration is equal among all beef types. Using likelihood-ratio tests, if the null is rejected, then further tests are needed to determine which of the beef types are statistically different from one another.

This second test is performed by using the estimated model to generate predicted values for U_k = noFTB_, U_k = 15FTB_, and U_k = maxFTB_, holding constant the order in which the samples are tasted, as well as demographic variables (which is why the *i* subscripts are suppressed). The predictions are made by assuming in (1) that *orderA* = *orderB* = *orderC* = 1/3, and that *female*, *age*, *eatburgers*, and *income50* are observed at their sample means of 0.62, 29.59, 0.77, and 0.64, respectively. Similar calculations are made for U_k = 15FTB_ and U_k = maxFTB_. To test the null hypothesis that U_noFTB_ = U_15FTB_, versus the alternative hypothesis that they are not equal, 50,000 parametric bootstraps following Krinsky and Robb [28] are employed. A 5% confidence level is used throughout, but after all statistical tests are conducted they will be adjusted to account for multiple testing.

### Second sensory evaluation: Sliders

While tasting bites of plain ground beef is the standard in sensory research, it has the disadvantage of being an unfamiliar and potentially unsatisfying eating experience. Few people eat plain ground beef, and so the first sensory evaluation is not representative of how people actually eat. With no salt or sauce, the patties may not provide much satisfaction, so subjects may have trouble rating differences in the three ground beef types because their minds are occupied with the dissatisfying taste. If a researcher wishes to know if regular consumers can tell a difference in their meals as finely textured beef (FTB) is added to ground beef, the first sensory evaluation leaves much to be desired.

To compensate for this weakness, a second evaluation is conducted immediately after the first, where subjects evaluate the three beef types in the context of a familiar eating experience. Subjects are given three different patties identified only by colored toothpicks: red, white, or blue (see Fig 1). Like the previous sensory evaluation, in which samples are identified by shapes, there are three different orderings of the colors used, with each color having roughly the same probability of being tasted first. Order A is red first, then white, then blue. Order B is white, blue, then red; Order C is blue, red, then white.

Each color refers to one of the three ground beef types studied (blue = no FTB, white = 15% FTB, and red = > 15% FTB), but this identification is not revealed to the researchers until the initial data analysis is complete. Once given the patties, individuals are also given three slider buns and are asked to build three sliders using whatever toppings they desire, making sure to keep their toothpicks on the same slider so that the identification of each beef product is preserved. The subjects are asked to keep the toothpicks inserted into their sliders throughout the evaluation so that they do not lose track of the slider’s identity.

Once given their sliders, the subjects proceed down a buffet line to build their burgers with whatever toppings they choose. The following toppings and condiments are available: salt, pepper, ketchup, mustard, BBQ sauce, lettuce, tomatoes, white onions, pickles, cheddar cheese, and mayonnaise. Although the subjects may use any combination of toppings, they are asked to make the three burgers as identical as possible, and researchers observe the burgers being built to ensure this is the case (see Fig 1). More than half of the subjects choose to add cheese and/or ketchup. Close to half add pickles, lettuce, and/or mustard. What results are three nearly identical sliders that differ only in the FTB content for any one subject, while the sliders differ by their FTB content and toppings across subjects. Side items such as potato chips and various drinks are also provided, but no other meats.

Each person is then asked to take one bite out of each slider and to rate its overall satisfaction using the same 9-Point Hedonic Scale (9 = like extremely … 1 = dislike extremely). Their questionnaires instruct them which slider to taste first and remind them to eat an unsalted cracker and take a sip of water between each bite. After one bite of each slider has been taken, they are then instructed to eat however much they like. Talking is now allowed and they are encouraged to enjoy their experience—again, to mimic how food is actually consumed. Once finished with their meal, they are asked to rate each slider once again using the same 9-Point Hedonic Scale, so that overall satisfaction with each slider can be evaluated both after the first bite of each slider as well as at the end of the meal.

Because only the attribute ‘overall satisfaction’ is used in this evaluation, all of the results can be summarized by the following ordered-logit model. This model has the same structure as (1), except that it also includes four additional variables, which allow the latent score to differ for each of the three beef types as well as any one beef type between the first bite and the end of the meal. These variables include *finishedmeal*, which equals zero if it is their first bite of the slider and one if they have finished the meal, and the other three new variables are interaction terms between *finishedmeal* and *NoFTB*, *15FTB*, and *maxFTB*. Note that the coefficient γ_2_ is normalized to equal zero for model identification.

$$\begin{array}{l} U_{i,k}=V_{i,k}\;+\;\tau_{i}+\epsilon_{ik}\\ =\left\{\beta_{1}\left(noFTB_{ik}\right)+\beta_{2}\left(15FTB_{ik}\right)+\beta_{3}\left(maxFTB_{ik}\right)\right\}\\ +\{\gamma_{1}\left(finishedmeal_{ik}\right)+\gamma_{2}\left(finishedmeal_{ik}\right)\left(noFTB_{ik}\right)\\ +\gamma_{3}\left(finishedmeal_{ik}\right)\left(15FTB_{ik}\right)+\gamma_{4}\left(finishedmeal_{ik}\right)\left(maxFTB_{ik}\right)\}\\ +\{\alpha_{1}\left(orderA_{ik}\right)+\alpha_{2}\left(orderB_{ik}\right)+\alpha_{3}\left(orderC_{ik}\right)+\alpha_{4}\left(female_{ik}\right)\\ +\alpha_{5}\left(age_{ik}\right)+\alpha_{6}\left(eatburgers_{ik}\right)+\alpha_{7}\left(income50_{ik}\right)\}+\tau_{i}+\epsilon_{ik} \end{array}$$ (3)

To test the null hypothesis that any two beef samples reflect identical scores, the null hypothesis that β_1_ = β_2_ = β_3_ = γ_1_ = γ_2_ = γ_3_ = γ_4_ = 0 is evaluated using a likelihood-ratio test. Also, predictions from the ordered logit model are used in conjunction with nonparametric bootstraps. For instance, to test whether subjects like sliders with no FTB more or less than sliders with 15% FTB, after the first bite, the value of U_k_ is calculated for both, and then the bootstraps are used to test the null hypothesis that they are equal, using the same procedure as in the first sensory evaluation.

### Third sensory evaluation: Hypothetical choice experiments

The previous sensory evaluations require the individuals to report their eating experiences using a hedonic scale. This can detect differences in preferences, but not how much more one beef might be preferred over the other. That is, it isn’t clear exactly what it means when one product is rated as ‘like moderately’ while another is rated ‘like slightly,’ other than the intuitive feel of the words. In these cases it is helpful to specify the preference differences in terms of dollars. Rating one product as ‘like moderately’ and another as ‘like slightly’ is useful information, but if it can be added that people say they will pay $0.25 more per pound for the former than the latter, the difference becomes more tangible.

This is accomplished by asking the respondents to answer four hypothetical choice experiments, shown in Fig 1, after they complete the first two sensory evaluations. For any one choice experiment, the subject is presented with the three beef products denoted as red, white, or blue (referring to the color of the toothpicks associated with each slider). The order in which the red, blue, and white burgers appear in the choice experiment is randomized across respondents, following the same order for presentation as in the previous evaluation. Each product is given a per pound price, and the subject is asked which product they would purchase in a grocery store. An option for ‘none’ is also available, which has a price of $0. The only differences in the four choice experiments are the prices. In one choice, the price of each ground beef product is $4.25 per pound, and in the remaining choices the price of two products is reduced to $3.50; therefore, each ground beef is listed as the more expensive product in one of the four choice experiments. This relatively simple experimental design, which follows that by Lusk and Schroeder [29], can be used because there are only four attributes that vary: the three beef products and the price. With a simple design that is the same across subjects, the results can be reported in tabular form, which allows readers unfamiliar with discrete choice statistical models to understand the results.

Note that because these are hypothetical choices they will be subject to hypothetical bias, where individuals say they will pay more for the meat than they really will. However, this study is not concerned with the overall willingness-to-pay for meat, but with the marginal willingness-to-pay for a different level of FTB, and studies have shown that while hypothetical choice experiments may overestimate total willingness-to-pay, they can accurately predict marginal willingness-to-pay [29].

The simplicity of the choice experiment allows us to display its results by simply showing the percentage of times each option is chosen in each of the four experiments. However, as with the hedonic scores, given the discrete nature of the data and the potential for demographic and ordering effects, a conditional logit model is used to test for statistical differences in preferences for the three beef types. Each choice experiment requires the respondent to select one option out of four (three ground beef products and a none option), and the option selected is assumed to provide them the highest degree of overall satisfaction, commonly referred to by economists as ‘utility,’ accounting for both the pleasure of eating the food as well as the displeasure of having to pay the listed price. The latent utility of each of the three beef types is assumed to follow a similar functional form as the hedonic scales, except that there is a variable for the price of the beef, and the error term unique to the individual and sample, ε_ik_, is assumed to follow a Type I Extreme Value Distribution, instead of the logistic distribution. Also, the impact of an individual error term, *τ*_*i*_, is accounted for using a fixed-effects conditional logit model using the clogit command in STATA.

$$\begin{array}{l} U_{ik}=V_{ik}\;+\;\tau_{i}+\epsilon_{ik}\\ =\left\{\beta_{1}\left(noFTB_{ik}\right)+\beta_{2}\left(15FTB_{ik}\right)+\beta_{3}\left(maxFTB_{ik}\right)\right\}\\ +\{\alpha_{1}\left(orderA_{ik}\right)+\alpha_{2}\left(orderB_{ik}\right)+\alpha_{3}\left(orderC_{ik}\right)+\alpha_{4}\left(female_{ik}\right)\\ +\alpha_{5}\left(age_{ik}\right)+\alpha_{6}\left(eatburgers_{ik}\right)\}+\alpha_{7}\left(income50_{ik}\right)\}-\rho \left(Price_{ik}\right)+\tau_{i}+\epsilon_{ik} \end{array}$$ (4)

If subjects are indifferent between the three slider types, then β_1_ = β_2_ = β_3_. This constitutes the null hypothesis, and the alternative hypothesis is that at least one of these coefficients is different from the others. A likelihood ratio test is conducted to calculate a p-value for this null hypothesis.

Any differences in the beef types can be quantified in the monetary or willingness-to-pay space. Note that if β_1_ > β_2_, the maximum premium the average individual will pay for ground beef with no FTB relative to beef with 15% FTB is given by (β_1_—β_2_)/ρ.

## Results

Recall that the objective of this research is to assess whether otherwise identical ground beef patties reflect different sensory properties to untrained subjects when the patties’ content of finely textured beef (FTB) varies from 0%, 15%, to some value greater than 15% FTB. Three different sensory evaluations are used, which ultimately entail a total of 27 hypothesis tests (four likelihood ratio and twelve nonparametric bootstrap tests from the first evaluation; one likelihood ratio and nine nonparametric bootstrap tests from the second evaluation; and one likelihood ratio test from the third evaluation). In all cases a significance level of 5% is used, meaning the null hypothesis is rejected if the p-value is less than 5%.

However, a single p-value is really only valid if it is the only test performed. The more statistical tests conducted, the greater the likelihood of rejecting a null hypothesis, even if the null is always true. Perform enough tests, and one is guaranteed to reject at least one null. Some means for correcting for multiple testing is thus needed. The method used here is the Benjamin and Hochberg method [27], which seeks to control the rate of false discoveries, or the rate of Type I Errors across multiple tests.

This method requires the researcher to rank all the p-values from the 27 tests (where 1 is the smallest p-value and 27 is the largest) and calculate a Benjamin-Hochberg test-statistic that equals (1/27)(targeted false discovery rate), where the targeted false discovery rate assumed here is 5%. The researcher then finds the largest p-value that is also less than its corresponding test-statistic, and that p-value and all smaller p-values are deemed statistically significant.

All of the above ordinal-logit and conditional-logit models are estimated using maximum likelihood with STATA/SE 14.2 for Mac, and all nonparametric bootstraps are performed in Matlab R2017A for Mac.

### First sensory evaluation results

**Fig 2:**
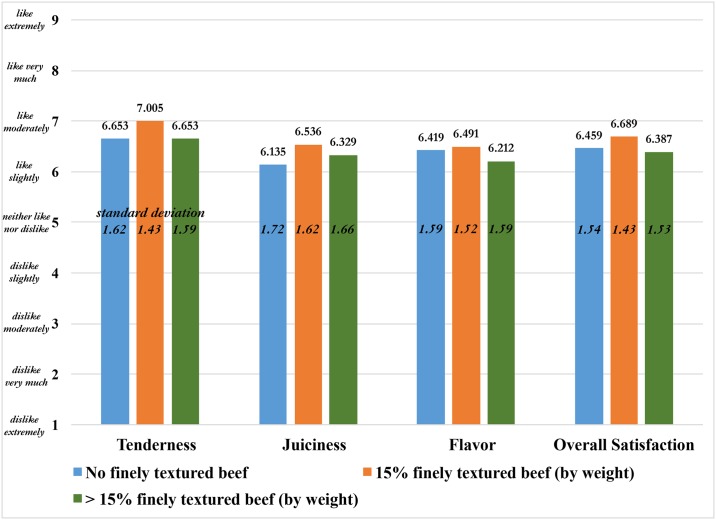
Average attribute ratings for plain ground beef samples with varying levels of finely textured beef (N = 222 untrained subjects).

The first sensory evaluation asks untrained subjects to rate the extent to which they like the tenderness, juiciness, flavor, and overall satisfaction of three plain beef samples that differ only in their percentage weight of FTB. The average ratings for the ground beef samples are shown in Fig 2, along with their standard deviations. All ratings are between the ‘like slightly’ to ‘like moderately’ range and appear relatively close in value. For each attribute, the beef sample with 15% FTB has a higher average than the other two samples, but Fig 2 alone is not sufficient for determining whether subjects really prefer one level of FTB over another.

Table 2 shows the ordered logit estimates for each attribute. Recall that the dependent variable is the latent (unobserved) predicted hedonic score, conditional on a set of explanatory variables. Though p-values for individual coefficients are often displayed in such tables, they are suppressed here because of the method used to correct for multiple testing later in the paper. Adding p-values to this table would reduce the information content of the p-values more pertinent to the study’s objectives. At the bottom of the table is the p-value for the null hypothesis that all three beef samples have the same mean score for the attribute of interest. The p-value for tenderness is 0.0099, considerably less than the threshold of 5%, suggesting that subjects do perceive a difference in at least two of the beef samples. The same can be said for juiciness, but not for flavor or overall satisfaction. So, while varying the level of FTB might influence the likeability of ground beef’s tenderness and juiciness, it does not alter the flavor-liking and does not necessarily lead to a more satisfying eating experience.

**Table 2:** Estimated coefficients of ordered logit model with random panel effects (plain, bite-size beef samples in first sensory evaluation).

	Attribute			
Variable	Tenderness	Juiciness	Flavor	Overall Satisfaction
noFTB (β_1_)	-------	-------	-------	-------
15FTB (β_2_)	0.4646	0.48640	0.0289	0.2836
maxFTB (β_3_)	-0.0093	0.24730	-0.3026	-0.1080
orderA (α_1_)	-0.1506	-0.41027	-0.1198	-0.3676
orderB (α_2_)	0.2683	-0.0818	-0.1876	-0.2237
orderC (α_3_)	-------	-------	-------	-------
female (α_4_)	0.0254	0.13060	-0.1017	0.1150
age (α_5_)	0.0097	0.0057	0.0097	0.0087
eatburgers (α_6_)	0.1805	0.26513	0.4403	0.6536
income50more (α_6_)	-0.1572	-0.41300	-0.2465	-0.3873
				
Threshold Parameters				
η_1_	-4.6589	-5.8077	-5.2275	-6.7770
η_2_	-3.0100	-4.4762	-3.2617	-4.7990
η_3_	-1.9555	-2.9608	-1.9698	-3.4082
η_4_	-1.4889	-1.7188	-1.3394	-1.9512
η_5_	-0.4067	-1.0080	0.0655	-1.3043
η_6_	1.1378	-0.02152	1.5961	-0.1011
η_7_	3.7862	1.5691	3.5921	1.6821
η_8_	-------	3.7250	-------	4.2157
σ	1.3440	1.3620	1.4454	1.3773
				
*P-value for null hypothesis that* β_1_ = β_2_ = β_3_ = 0	*0*.*0099*	*0*.*0209*	*0*.*1105*	*0*.*0729*

To identify any latent constructs influencing the attribute ratings, a factor analysis of all four attributes and all three beef types is conducted using the factanal routine in R using varimax-rotation. Chi-square tests suggest a total of seven factors exist, with their factor loadings reported in Table 3. The first three factors seem to load heavily on one of the three beef types. Factor 1 reveals correlations in attribute ratings for the beef with no FTB. Likewise, Factors 2 and 3 denote latent constructs corresponding to the 15% FTB and max FTB beef types, respectively. These three factors suggest that the attribute ratings within any one beef type are positively correlated. The interpretations of the remaining factors are less clear, but might signify correlations for certain attributes across beef types. Factor 4 has loadings for all three tenderness ratings, Factor 6 has loadings for all juiciness ratings, and Factor 7 has loadings for all overall satisfaction ratings.

**Table 3:** Factor analysis of attribute ratings for three levels of finely textured beef.

Variable	Factor 1	Factor 2	Factor 3	Factor 4	Factor 5	Factor 6	Factor 7
Tenderness: no FTB	**0.8330**^a^			**0.1680**			
Flavor: no FTB	**0.7980**	0.1580	0.2120				
Juiciness: no FTB	**0.8030**	0.1410	0.1090			**0.2190**	
Overall: no FTB	**0.9720**	0.1320	0.1320				**0.1170**
Tenderness: 15% FTB	0.2110		**0.6520**	**0.6420**			
Flavor: 15% FTB	0.1700	0.1010	**0.8610**			-0.1140	-0.1190
Juiciness: 15% FTB	0.1800	0.1020	**0.7370**	0.1420		**0.5830**	
Overall: 15% FTB	0.1460	0.1200	**0.9280**	0.1190		0.1010	**0.1260**
Tenderness: > 15% FTB	0.1660	**0.8150**		**0.1200**	-0.1420		-0.1880
Flavor: > 15% FTB	0.1180	**0.7400**	0.1610		0.6380		
Juiciness: > 15% FTB	0.1450	**0.8170**				**0.1030**	
Overall: > 15% FTB	0.1290	**0.9350**	0.1100		0.1810		**0.1960**
							
Sum of Squared Factor Loadings	3.1270	2.8640	2.703	0.5050	0.4730	0.4350	0.1230
Cumulative Variance Explained by Factors	0.2610	0.4990	0.7240	0.7670	0.8060	0.8420	0.8520

^a^ Items in bold are meant to highlight possible meanings behind the seven latent constructs.

**Fig 3:**
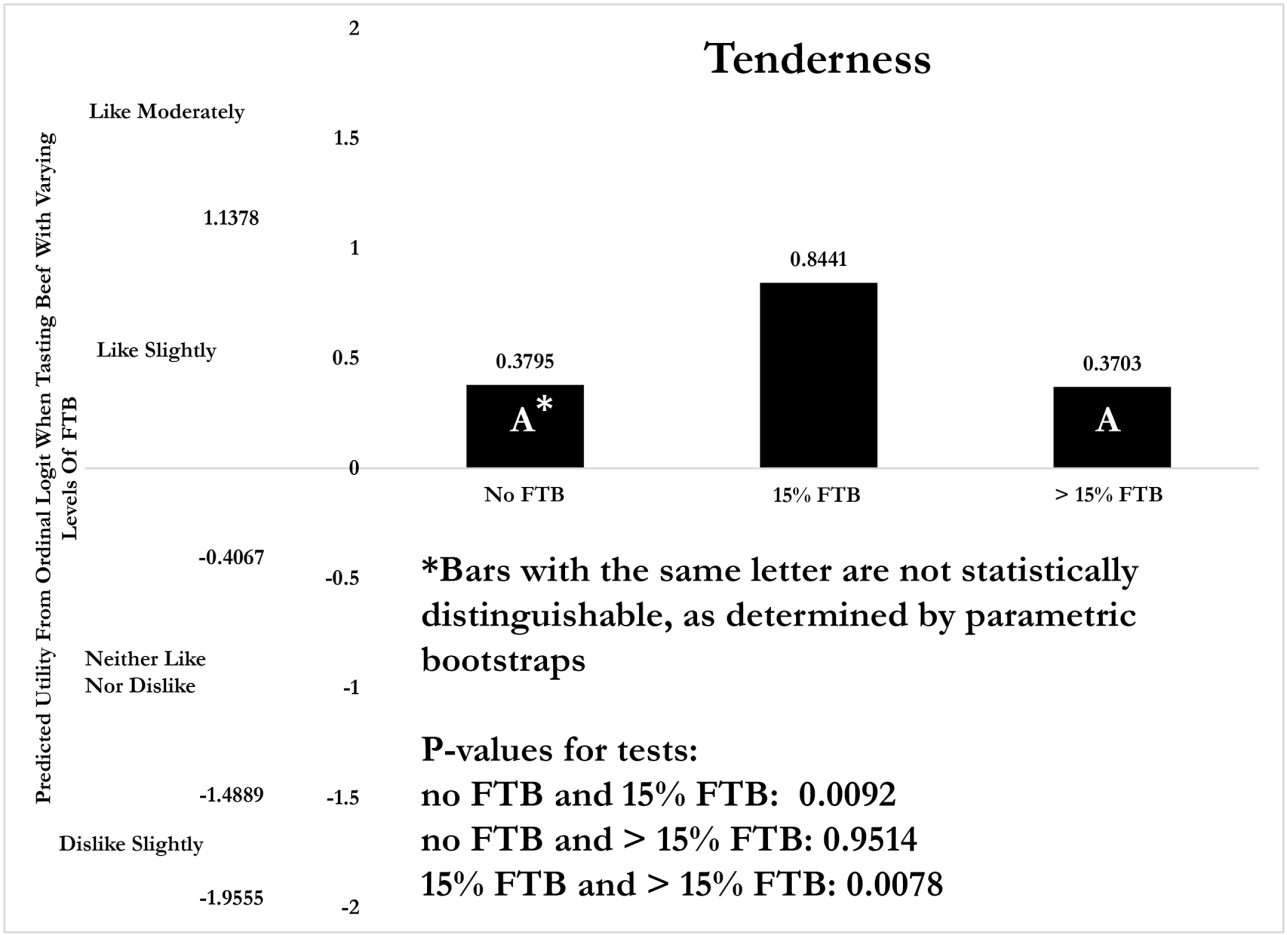
Predicted latent hedonic scores of tenderness attribute in plain ground beef samples. Predicted scores assume that orderA = orderB = orderC = 1/3 and that other explanatory variables are observed at their samples means. Parametric bootstraps are used to generate p-values for the null hypothesis that the predicted score for any one beef sample is equal to the predicted score of another sample.

Next, an investigation is performed to determine which of the three ground beef products have a more favorable tenderness and juiciness rating. The predicted score for, say, tenderness is obtained by assuming OrderA = OrderB = OrderC = 1/3, thereby neutralizing any order effect, and then assuming the other explanatory variables are observed at their sample means. The results are shown in Fig 3, which illustrates the predicted score relative to the threshold parameters. All predicted scores are greater than -0.4067 and less than 1.1378 (two threshold parameters in Table 2), which puts the scores in the range ‘like slightly.’ This is roughly the same score as that provided in Fig 1, suggesting that correcting for ordering and demographic effects does not alter the hedonic scores for tenderness much. This is not surprising, as the order in which the samples are tasted are randomized in the experiment, and previous studies have shown demographics to play only a minor role in hedonic scores for beef [25].

Fig 3 shows that the tenderness scores for beef with no FTB and with more than 15% FTB are not statistically distinguishable, indicating the subjects prefer the tenderness of both equally. The sample with 15% FTB has a higher and statistically significant score, suggesting that subjects do indeed prefer the tenderness of ground beef containing 15% FTB. Note that this doesn’t mean the 15% FTB sample is more tender, only that subjects *like* its tenderness more. Thus, adding FTB to ground beef can perhaps improve its tenderness-likeability, but more is not always better.

**Fig 4:**
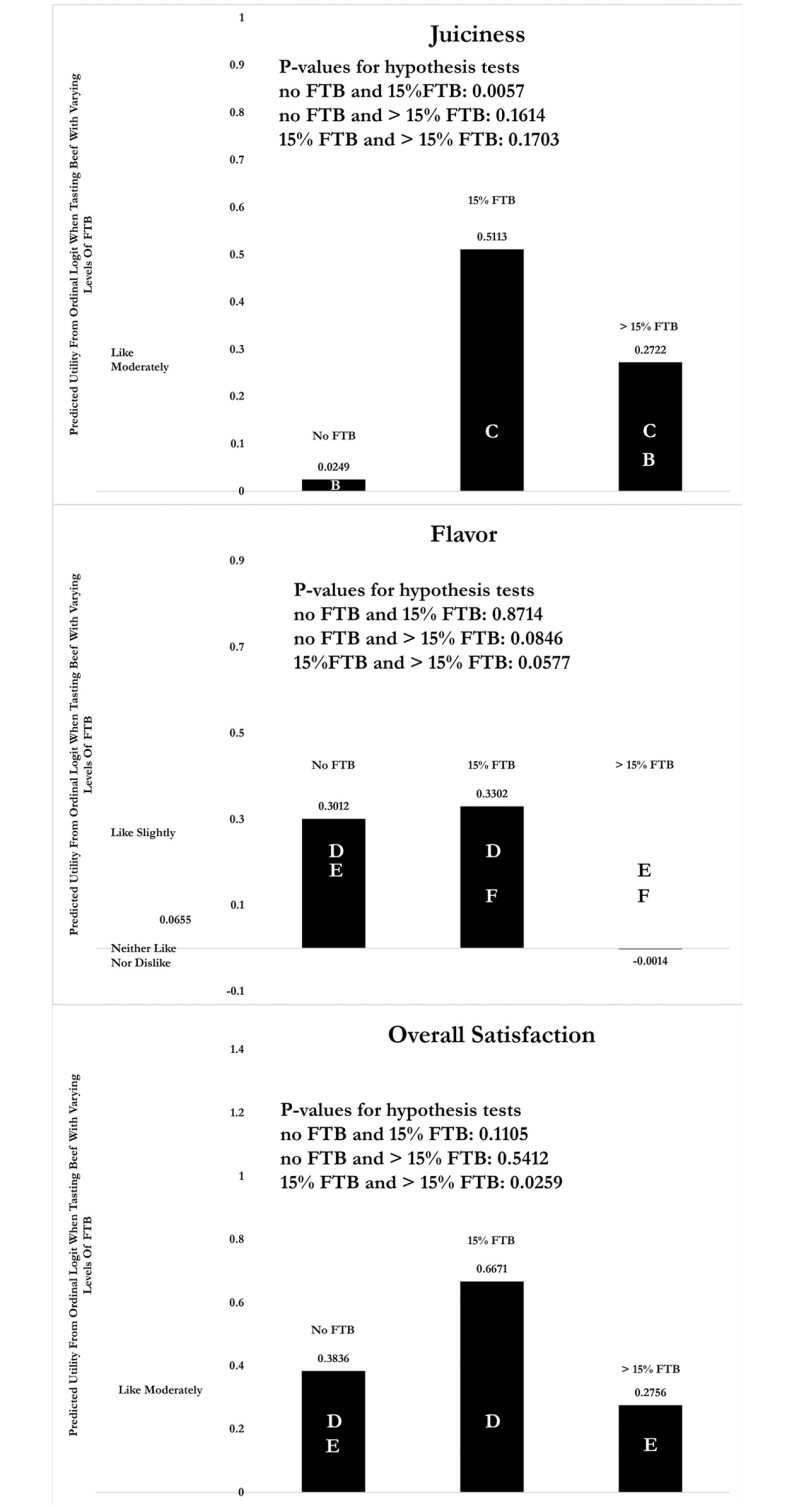
Predicted latent hedonic scores of juiciness, flavor, and overall satisfaction attribute in plain ground beef samples. Predicted scores assume that orderA = orderB = orderC = 1/3 and that other explanatory variables are observed at their samples means. Parametric bootstraps are used to generate p-values for the null hypothesis that the predicted score for any one beef sample is equal to the predicted score of another sample.

Similar Figs are constructed for juiciness, flavor, and overall satisfaction and are shown in Fig 4. In all cases, beef with 15% FTB has a higher predicted score, but in only two cases are the differences statistically significant. In terms of juiciness, beef with 15% FTB is more likeable than beef with no FTB, but it is not different than beef with more than 15% FTB. However, in regard to flavor, all three beef samples are statistically indistinguishable from one another, unless one is willing to increase the confidence level from 5% to 6%. In terms of overall satisfaction, beef with 15% FTB generates more overall satisfaction than beef with more than 15% FTB, but it is roughly the same as beef with 0% FTB.

### Second sensory evaluation results

The previous results pertain only to plain, bite-size pieces of ground beef patty—a very alien eating experience to most subjects, but the second sensory evaluation compares the beef samples in a more realistic eating experience. Subjects make virtually identical sliders with three patties, each of which varies only by its content of finely textured beef (FTB). As in the previous section, the subjects evaluate their overall satisfaction with each slider on the 9-point Hedonic Scale.

**Fig 5:**
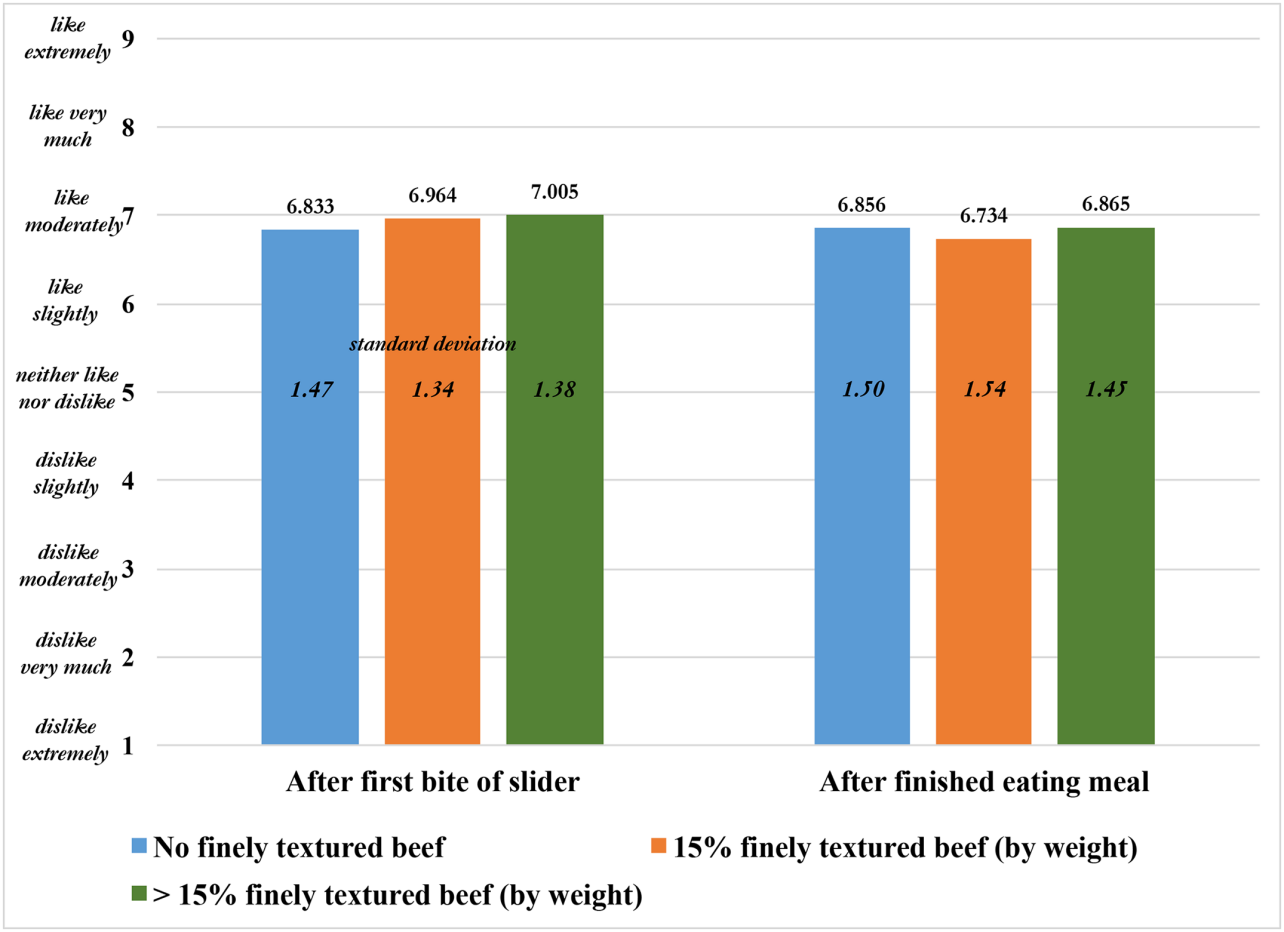
Average attribute ratings for sliders (small hamburgers) made from ground beef patties with varying levels of finely textured beef (N = 222 untrained subjects).

The raw results, not conditional on specific demographics or ordering effects, are shown in Fig 5. Given there is little difference in overall satisfaction for plain ground beef patties in the previous section, one would expect little difference in satisfaction from sliders, where the ground beef is evaluated concomitant with the buns and condiments. Indeed, Fig 5 suggests this is the case, with each beef type receiving an average score roughly equal to ‘like moderately.’ This is the case both when the beef is evaluated after the first bite of the slider and when the meal is complete.

To evaluate the predicted latent score of the three sliders based on common demographics and holding constant ordering effects, the estimates of the ordered logit model are given in Table 4 below. The last row of the table tests the null hypothesis that each slider, regardless of its FTB content or when it is evaluated, provides an identical latent score, and its large p-value of 0.54 suggests subjects like sliders made from all three beef types equally.

**Table 4:** Estimated coefficients of ordered logit model with random panel effects (beef patties in custom-made sliders in second sensory evaluation).

Variable	Attribute Overall Satisfaction
noFTB (β_1_)	-------
15FTB (β_2_)	0.2083
maxFTB (β_3_)	0.3588
finishedmeal (γ_1_)	0.1031
noFTB*finishedmeal (γ_2_)	-------
15FTB*finishedmeal (γ_3_)	-0.3760
maxFTB*finishedmeal (γ_4_)	-0.3456
orderA (α_1_)	-0.3545
orderB (α_2_)	-0.2106
orderC (α_3_)	-------
female (α_4_)	-0.2377
age (α_5_)	-0.0047
eatburgers (α_6_)	0.4510
income50more (α_6_)	-0.1079
	
Threshold Parameters	
η_1_	-7.4485
η_2_	-6.1798
η_3_	-4.5748
η_4_	-3.1353
η_5_	-2.3009
η_6_	-1.0993
η_7_	0.5113
η_8_	3.0038
σ	1.8595
	
*P-value for null hypothesis that* β_1_ = β_2_ = β_3_ = γ_1_ = γ_2_ = γ_3_ = γ_4_ = *0*	*0*.*5383*

Of course, it could be that two sliders really are different, but the stochastic noise across all tastings makes it hard to distinguish, so nonparametric bootstraps are used to conduct more specific statistical tests. Table 5 shows the p-values for the null hypothesis that two beef samples provide the same overall satisfaction. For instance, the p-value of 0.24 suggests that the same latent score is assigned to sliders with no FTB after the first bite, as is assigned to sliders with 15% FTB, also evaluated after the first bite. The lower p-value of 0.05 suggests that consumers might not be indifferent between a slider with no FTB and a slider with > 15% FTB, both after the first bites. (However, once multiple testing is accounted for, this p-value may no longer be statistically significant.) The p-value of 0.17 in the lower right-hand side argues that subjects like the slider with > 15% FTB the same, regardless of whether they are evaluating it after the first bite or after the meal is complete. Overall, the p-values in Tables 4 and 5 suggest there are little to no differences in the sliders with different levels of FTB—or if there are, subjects like them all equally.

**Table 5:** P-Values for the null hypothesis that hedonic scores for overall satisfaction between two slider tastings are the same (beef patties in custom-made sliders in second sensory evaluation).

Sample A	Sample B		
	No FTB: First Bite	15% FTB: First Bite	> 15% FTB: First Bite
No FTB: First Bite	-----	-----	-----
15% FTB: First Bite	0.24	-----	-----
> 15% FTB: First Bite	0.05	0.39	-----
	No FTB: Second Bite	15% FTB: Second Bite	> 15% FTB: Second Bite
No FTB: Second Bite	-----	-----	-----
15% FTB: Second Bite	0.34	-----	-----
> 15% FTB: Second Bite	0.94	0.31	-----
	No FTB: First Bite	15% FTB: First Bite	> 15% FTB: First Bite
No FTB: Second Bite	0.56	-----	-----
15% FTB: Second Bite	-----	0.12	-----
> 15% FTB: Second Bite	-----	-----	0.17

Notes: p-values are calculated from nonparametric bootstraps of the predicted latent scores of the ordinal-logit regression estimates in Table 4.

These results only refer to average preferences. Could it be that some people just prefer beef with no FTB while others prefer beef with 15% FTB, such that the aggregate sample gives the appearance of indifference? This could be the case if half of the subjects prefer no FTB in both evaluation treatments and half prefer 15% in both evaluation treatments, but if many subjects express an inconsistency between the beef type they prefer between the two treatments, it would suggest a true indifference between the levels of FTB.

Of the 222 subjects, when comparing no FTB to 15% FTB, 62% express inconsistent preferences, either preferring no FTB for plain beef samples and 15% FTB in sliders, or vice-versa, so most of the subjects choose different beef products in the two evaluations. Though it is possible that the actual likeability of FTB in beef truly depends on the context in which it is eaten (plain beef versus sliders) and though some subjects may truly prefer a particular level of FTB, the bulk of the evidence suggests an indifference for beef with and without FTB across most subjects when the level of FTB varies from 0%, to 15%, or to some specific but unknown level greater than 15%.

### Third sensory evaluation results

Given that the subjects assign similar hedonic scores to each of the three beef types in the slider evaluation, one would not expect consumers to be willing to pay a higher price for one beef type than another or to prefer one particular beef type to another at the same price. Thus, the results in Table 6 are not surprising.

**Table 6:** Selections made by respondents in choice experiments.

Choices	Slider 0% FTB	Slider 15% FTB	Slider > 15% FTB	No Slider
	*Percentage of subjects who select each of the four options (one of three sliders or no slider) under each hypothetical choice set*			
***Choice Set 1***				
**No FTB: $4.25** **15% FTB: $4.25** **Max FTB: $4.25** **None: $0.00**	27%	29%	30%	14%
***Choice Set 2***				
**No FTB: $4.25** **15% FTB: $3.50** **Max FTB: $3.50** **None: $0.00**	9%	45%	41%	5%
***Choice Set 3***				
**No FTB: $3.50** **15% FTB: $4.25** **Max FTB: $3.50** **None: $0.00**	40%	12%	40%	8%
***Choice Set 4***				
**No FTB: $3.50** **15% FTB: $3.50** **Max FTB: $4.25** **None: $0.00**	44%	39%	12%	5%

This table shows the percentage of subjects who choose each beef product in each choice set (recall each subject faces the same four choice sets). Choice Set 1 shows that when all beef types are sold at the same price, each beef type is purchased at roughly the same rate: 27–31%. When one particular beef is sold at a lower price, its hypothetical purchases rise and the percentage who select ‘none’ falls, but the two remaining beef types at the same price are still purchased at roughly the same rate. The table demonstrates that consumers are paying attention to the question, making what seem like rational choices in that they are responding to price, but it also demonstrates that they are indifferent between each beef type.

Still, it should be noted that around 9–13% of subjects are willing to pay a higher price for one particular beef type, which means that as an individual they believe one level of FTB really is superior. Though the results overall suggest that, in aggregate, consumers are indifferent between the three levels of FTB, some specific consumers are not literally indifferent and are willing to pay up to 20% more for their preferred slider. However, given that only around 10% of the subjects seem to truly believe one slider is better, the overall consensus is that subjects in general are indifferent between the three levels of FTB considered.

To test whether subjects are still indifferent between the three levels of FTB when demographics and the order in which the sliders are tasted are held constant, Table 7 reports the results of a conditional logit model for the selections made in the choice experiment. This model differs from the ordinal-logit model in that it predicts not a latent hedonic score, but the latent utility expected from being able to purchase each beef type at the listed price. The p-value at the bottom of the table tests whether subjects are indifferent, on average, between the three beef types. Its high value of 0.82 further confirms that one could vary the FTB in sliders between the three levels considered here and the subjects, again, on average, would prefer each equally.

**Table 7:** Estimated coefficients of conditional logit model with fixed panel effects (choice experiments).

Variable	Coefficient
	
noFTB (β_1_)	6.5121
15FTB (β_1_)	6.4949
maxFTB (β_1_)	6.5900
Price (ρ)	-1.2594
orderA (α_1_)	-0.4502
orderB (α_2_)	-0.4291
orderC (α_3_)	-------
female (α_4_)	0.5285
age (α_5_)	-0.0266
eatburgers (α_6_)	0.9825
income50more (α_6_)	0.3213
*P-value for null hypothesis that* β_1_ = β_2_ = β_3_ = 0	*0*.*8204*

### All tests considered

Although some of the aforementioned tests have p-values less than 5%, suggesting statistical significance, because a total of twenty-seven tests are conducted it is unclear whether a low p-value occurs because the null hypothesis is false, or simply due to randomness. With multiple tests, the probability of committing a Type I Error when using the 5% significant level is in fact greater than 5%. To help put the p-values in perspective, the Benjamin-Hochberg method [27] is used to evaluate the tests.

This test requires the researcher to rank all 27 p-values, where 1 = smallest and 27 = largest (let this ranking be *r* and the total number of tests be *T*). The researcher must then specify a targeted false discovery rate (*FDR*), which is akin to a Type I Error but refers to false discoveries across tests as opposed to within a test. This is the maximum number of times the researcher wants to reject a null hypothesis that is in fact true across tests. A Benjamin-Hochberg critical value is computed for each p-value as (*r*/*T*)(*FDR*). The largest p-value that is less than (*r*/*T*)(*FDR*), as well as all p-values smaller (regardless of whether they are less than their corresponding critical value), are then deemed statistically significant.

The complete listing of the 27 p-values and their critical values using a false discovery rate of 5% and 10% is shown in S1 Table, and the five smallest p-values are shown in Table 8. These tables show that under a 5% false discovery rate, every one of the 27 p-values are in fact not statistically significant. Under this rate, then, one must say that there are zero differences in the likeness of any of the beef samples for any of the attributes or evaluations. However, if a higher rate of 10% is used, four p-values become significant. With this higher rate one can confirm that when consuming plain, bite-size beef samples, consumers do like the tenderness and juiciness of some samples more than others: the beef sample with 15% FTB has a more desirable tenderness rating than no FTB and > 15% FTB, and the beef with 15% FTB has a more desirable juiciness rating than beef with no FTB. Despite these differences, when consumed in the form of a slider, subjects are indifferent between the three levels of FTB.

**Table 8:** Using the Benjamin-Hochberg method to correct for multiple testing (showing only the lowest five p-values).

Sensory Evaluation	Type of Statistical Test	Description of Null Hypothesis	P-Value of Null	Benjamin-Hochberg Critical Values	
				5% False Discovery Rate	10% False Discovery Rate
1st sensory evaluation: juiciness of plain beef	parametric simulations	same latent juiciness score for no FTB and 15% FTB	0.0057	0.0019	**0.0037**
1st sensory evaluation: tenderness of plain beef	parametric simulations	same latent tenderness score for 15% FTB and >15% FTB	0.0078	0.0037	**0.0074**
1st sensory evaluation: tenderness of plain beef	parametric simulations	same latent tenderness score for no FTB and 15% FTB	0.0092	0.0056	**0.0111**
1st sensory evaluation: tenderness of plain beef	likelihood-ratio test	coefficients in ordinal-logit same for all beef types	0.0099	0.0074	**0.0148**
1st sensory evaluation: juiciness of plain beef	likelihood-ratio test	coefficients in ordinal-logit same for all beef types	0.0209	0.0093	0.0185

The consensus of the tests then is that there is weak evidence for the claim that ground beef with some amount of finely textured beef might have a more favorable tenderness and juiciness rating than beef with none and that more of the finely textured beef is not always better. In relation to flavor and overall satisfaction, however, the evidence suggests there is no difference among beef with varying levels of finely textured beef—at least within the levels considered in this research.

## Discussion

There are reasons for expecting a more favorable tenderness or juiciness rating for beef with finely textured beef (FTB). For example, the addition of FTB can increase the pH of ground beef patties. A greater pH can shift the isoelectric point of meat, thus allowing meat proteins to hold more water [30]. Similarly, a greater pH can solubilize meat proteins. Hence, this can lead to less water loss after cooking, resulting in more juiciness. In support, Moon *et al*. [3] reported lower instrumental shear force with the addition of more FTB in ground beef patties. Previous studies utilizing beef strip loins enhanced with ammonium hydroxide reported improved tenderness. Further, trained taste panel ratings revealed an increase in tenderness, a decrease in connective tissue, and an increase in juiciness when beef chuck was enhanced with ammonium hydroxide and salt [31, 32]. Steaks enhanced with ammonium hydroxide at the 1% level demonstrated more beef flavor than control non-enhanced steaks [33]. Mechanical processes involved in FTB, such as grinding, can improve the tenderness of FTB due to physical breakdown of protein and connective tissue.

The similarity of the three products studied could be due to the fact that the samples eaten are not particularly desirable. The unappealing nature of an unseasoned, plain ground beef burger is evidenced by the fact that it is rarely eaten in such a fashion. It might be difficult for subjects to rate the extent to which they like attributes more in one sample compared another when they don’t like any of the samples. This is why a sensory analysis of a realistic eating experience provides particularly useful information—but it also limits the generalizability of the results, as they refer to sliders/hamburgers only.

Perhaps different results would be found if the meal consisted of chili or tacos? This seems unlikely, for if FTB has any impact it is to make the beef more tender. Both chili and tacos use crumbled ground beef simmered in liquid, which acts to tenderize the meat. Even if the ground beef remains a patty but is cooked in the fashion of Salisbury steak, the simmering would tenderize the beef. This suggests that tacos/chili/Salisbury steak would be tender regardless of the FTB level. This is just a reasoned conjecture, though; experiments are needed to verify whether it is the case.

Another factor limiting the generalizability of these results is that subjects are not informed of the level of FTB in each ground beef sample, nor is FTB or any other attribute even mentioned. If FTB is increasingly returning to the ground beef market, but another news story similar to those in 2012 arises, this study cannot be used to predict preferences for FTB in beef. The blind taste tests allow only analysis of experience attributes of the beef, yet given the perceived safety concerns of FTB, the meat also possesses credence attributes that could cause consumers to prefer less FTB. Indeed, the media coverage in 2011–2012 was largely about credence attributes.

Preferences for beef incorporate many credence attributes, such as how and where the cattle are raised and whether the meat is traceable back to the farm. The greater the consumption of beef, the more complex the role these credence attributes become, and many of them relate to food safety [34]. If consumers feel the FTB process leads to riskier and unhealthier food, or if they simply find the description of the process unappealing, they may lower their demand for ground beef containing it, regardless of whether the taste stays the same and their experienced health is unimpaired.

Moreover, experience and sensory attributes can interact with one another, as information about a product that does not affect experience attributes in blind taste tests may actually alter the taste when the blindfold is lifted. Studies have shown that identical products can receive different sensory scores when people are given different information about the products, such as if the meat is said to be raised humanely [35] or if tomatoes are said to be grown organically [36], regardless of whether those statements are true. Even the ambient noise at the time of tasting can influence the intensity of sweet sensations in food [37]. It is possible that knowing ground beef contains something referred to in the media as ‘pink-slime’ would not only make subjects less likely to purchase the beef, but would also change their sensory perceptions of the beef.

## Conclusions

The beef industry has argued that consumers prefer meat containing some amounts of finely textured beef (FTB), but these claims are from private sources and have not been verified objectively [9,10]. Given that FTB was such a public issue in years past, it is important that claims regarding its advantages and disadvantages be tested scientifically. Although ground beef containing 15% FTB by weight might provide a more favorable tenderness and juiciness profile over 0% FTB when consumed as plain, bite-size pieces, consumers do not prefer it when evaluating its overall satisfaction, especially when consuming it in the form of sliders. Although the evidence for differences in sensory properties in ground beef containing varying levels of FTB is weak, it is perhaps strong enough to warrant further research.

## Supporting information

S1 Appendix Sample questionnaire. Sample questionnaire given to subjects.
(DOCX)

S1 Table Using the Benjamin-Hochberg method to correct for multiple testing (all 27 tests). (DOCX)
