# Supplementary material for: Preference evaluation of ground beef by untrained subjects with three levels of finely textured beef
Source: PLoS One. 2018 Jan 17;13(1):e0190680. doi: 10.1371/journal.pone.0190680 (PMC5771606; doi:10.1371/journal.pone.0190680)
Supplement: S1 Appendix — Sample questionnaire given to subjects. (DOCX) [file pone.0190680.s001.docx]

**S1 Appendix. Full survey.** Note that due to the randomization of which shapes and colors subjects were asked to try first, not all surveys are identical.

**PARTICIPANT INFORMATION**

**OKLAHOMA STATE UNIVERSITY**

**Project Title: Preferences for ground beef**

**Purpose:** The objective of the research is to study people’s preferences for ground beef. You must be 18 or older to participate.

**What to Expect:** To participate in this study you must be willing to taste ground beef and hamburgers and provide feedback on your eating experience. First you will be asked to taste three pieces of ground beef and report your preference. Then you will be given three sliders (small hamburgers) and asked to make three nearly-identical hamburgers, including whatever toppings and condiments you wish. You will then report your preference for the burgers. All food has been prepared by a meat scientist and so will be as safe as a normal meal.

When you are done eating you will be given $10 for your participation.

**Risks:** There are no risks associated with this project which are expected to be greater than those ordinarily encountered in daily life. At no point do we ask your contact information, so your identity cannot be matched with your responses.

**Benefits:** A chance to help researchers understand your preferences for ground beef.

**Compensation:** A free meal and $10 in cash.

**Your Rights and Confidentiality:** Your participation in this research is voluntary. There is no penalty for refusal to participate, and you are free to withdraw your consent and participation in this project at any time. If you feel you may have an allergy to any of the foods, please let the researchers know promptly, and you may cease participating with no penalty.

**Confidentiality:** You will be given an identification number and at no time will you be asked for your contact information. Thus, it would be impossible for anyone to match your responses to your identity.

**Contact:** You may contact any of the researchers at the following addresses and phone numbers, should you desire to discuss your participation in the study and/or request information about the results of the study:

Bailey Norwood. 426 Ag Hall. Department of Agricultural Economics. Oklahoma State University. 405-334-0010. [bailey.norwood@okstate.edu](mailto:bailey.norwood@okstate.edu). fbaileynorwood.com.

If you have questions about your rights as a research volunteer, you may contact the IRB Office at 219 Cordell North, Stillwater, OK 74078, 405-744-3377 or irb@okstate.edu

**CONSENT DOCUMENTATION:**

I have been fully informed about the procedures listed here. I am aware of what I will be asked to do and of the benefits of my participation. I also understand the following statements:

I affirm that I am 18 years of age or older.

| *Preface the signature lines with the following statement (expand if appropriate):*  I have read and fully understand this consent form. I sign it freely and voluntarily. A copy of this form will be given to me. I hereby give permission for my participation in this study.  _________________________________________ _________________________  Signature of Participant Date  I certify that I have personally explained this document before requesting that the participant sign it.  _________________________________________ _________________________  Signature of Researcher Date |
| --- |

**Instructions for subjects**

- Please sit anywhere you like. This session will proceed as follows.
- **Part A:** First we will bring you each three pieces of ground beef. After taking each bite, please cleanse your palate by eating a cracker and taking a sip of water. You will taste each piece and answer a few questions about your eating experience.
- **Part B:** Then you will be given three sliders (small hamburgers) and will be asked to build identical hamburgers using whatever toppings you wish. You may also take whatever side dishes and drinks you wish. You will take one bite from each slider and report your eating experience. Between each bite, please cleanse your palate by eating a cracker and taking a sip of water. As you eat, please do not talk amongst each other about the burgers or the beef. After taking one bite of each burger and reporting your experience, you are free to continue eating and socializing, and you may talk about anything except the beef and burgers.
- **Part C:** After you have finished eating you will indicate once again your eating experience.
- **Part D:** You will indicate which ground beef products you would purchase at various prices.
- **Part E:** You will comment on whether you believe the burgers are identical or different from each other.
- **Part F:** You will answer a few questions about yourself.

***(A) Meats labeled square, triangle, and circle***

| Please indicate the extent to which you like or dislike the tenderness, flavor, juiciness, and overall satisfaction of the beef labeled *square*. | | |
| --- | --- | --- |
| **SQUARE** | **Tenderness** | 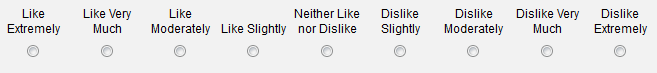 |
|  | **Flavor** | 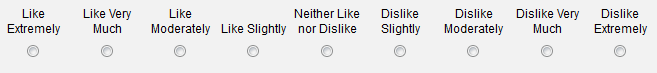 |
|  | **Juiciness** | 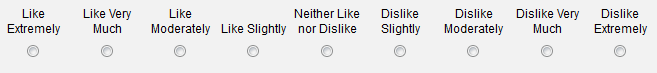 |
|  | **Satisfaction with overall eating quality** | 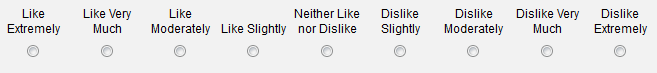 |

*(Remember to cleanse your palate by eating a cracker and taking a sip of water.)*

| Please indicate the extent to which you like or dislike the tenderness, flavor, juiciness, and overall satisfaction of the beef labeled *triangle.* | | |
| --- | --- | --- |
| **TRIANGLE** | **Tenderness** | 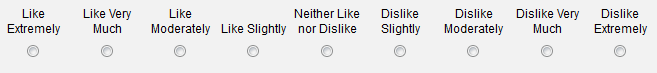 |
|  | **Flavor** | 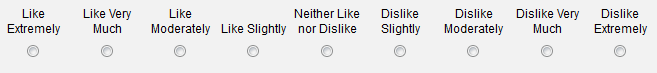 |
|  | **Juiciness** | 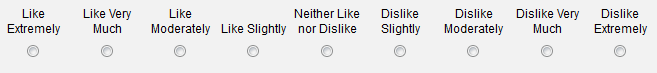 |
|  | **Satisfaction with overall eating quality** | 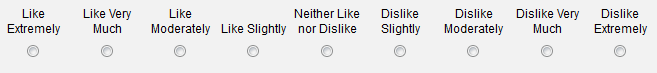 |

*(Remember to cleanse your palate by eating a cracker and taking a sip of water.)*

| Please indicate the extent to which you like or dislike the tenderness, flavor, juiciness, and overall satisfaction of the beef labeled *circle.* | | |
| --- | --- | --- |
| **CIRCLE** | **Tenderness** | 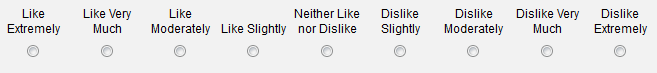 |
|  | **Flavor** | 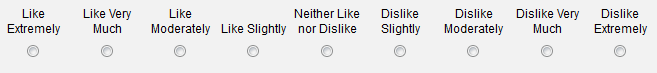 |
|  | **Juiciness** | 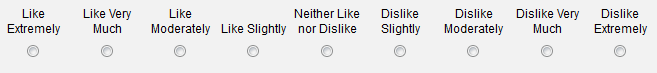 |
|  | **Satisfaction with overall eating quality** | 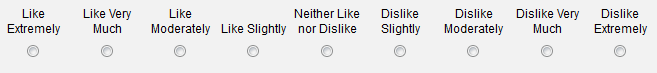 |

When you have finished Part A you may then build three identical sliders (small hamburgers) and take whatever side-dishes and drinks you like.

***(B) Burgers labeled red, white, and blue (first bites)***

| Using the sliders labeled red, white, and blue, make identical burgers using the same toppings and in the same amount. Take one bite from each slider and then indicate below the extent to which you like the overall eating experience.  *(Remember to cleanse your palate by eating a cracker and taking a sip of water between each bite.)* | |
| --- | --- |
| Red | 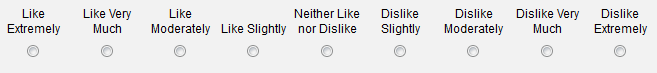 |
| White | 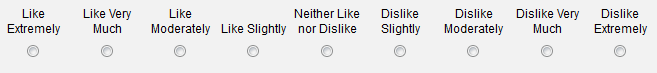 |
| Blue | 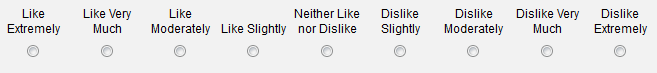 |

After you have finished your meal please complete all remaining questions.

***(C) Burgers labeled red, white, and blue (after you are finished)***

| Now that you have finished eating, please indicate below the extent to which you like the overall eating experience. | |
| --- | --- |
| Red | 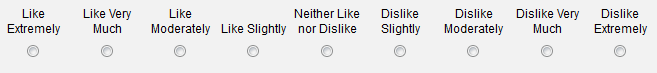 |
| White | 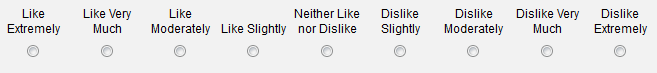 |
| Blue | 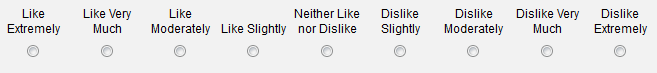 |

***(D) Food purchasing decisions***

Imagine you are in the grocery store buying a package of ground beef. There are three ground beef options exactly the same as the options you tried today: red, white, and blue. For each of the following four questions that follow, please indicate which option you would be most likely to buy.

**Which of the following would you purchase?**

| *Choice #1* | Red  $4.25/lb | White  ​$4.25/lb | Blue  ​$4.25/lb | If these were the only options, I would buy something else. |
| --- | --- | --- | --- | --- |
| I would choose... |  |  |  |  |

| *Choice #2* | Red  $3.50/lb | White  ​$3.50/lb | Blue  ​$4.25/lb | If these were the only options, I would buy something else. |
| --- | --- | --- | --- | --- |
| I would choose... |  |  |  |  |

| *Choice #3* | Red  $3.50/lb | White  ​$4.25/lb | Blue  ​$3.50/lb | If these were the only options, I would buy something else. |
| --- | --- | --- | --- | --- |
| I would choose... |  |  |  |  |

| *Choice #4* | Red  $4.25/lb | White  ​$3.50/lb | Blue  ​$3.50/lb | If these were the only options, I would buy something else. |
| --- | --- | --- | --- | --- |
| I would choose... |  |  |  |  |

**(E) What were these three products?**

The three ground beef products may be different or they may be identical. If you believe they are different, can you speculate on how they are different?

_____ I think the red, white, and blue products are identical

_____ I think at least two of the products are different (*Please speculate in the box below how you think they are different. Are they cooked differently? Made from different types of meat? Any thoughts you have are welcome*.)

**(F) A few more questions**

(F.1) Please check all toppings and condiments you placed on your burgers. *Please check all that apply.*

| □ ketchup | □ pickles |
| --- | --- |
| □ mustard | □ cheddar cheese |
| □ BBQ sauce | □ mayonnaise |
| □ lettuce | □ bun |
| □ tomatoes |  |
| □ white onions |  |

(F.2) What is your gender? *Please check one.*

| □ male | □ female | □ other |
| --- | --- | --- |

(F.3) What is your age? ________ years

(F.4) How often do you eat hamburgers? *Please check one.*

| □ Frequently | □ Rarely | □ Never |
| --- | --- | --- |

(F.5) How often do you eat ground beef in the form of any food (for example, hamburgers, tacos)? *Please check one.*

| □ Frequently | □ Rarely | □ Never |
| --- | --- | --- |

(F.6) How often do you or your household purchase ground beef? *Please check one.*

| □ At least once a week |  |
| --- | --- |
| □ At least once every two weeks |  |
| □ At least once a month |  |
| □ At least once every two months |  |
| □ Less than once every two months |  |
| □ Never |  |

(F.7) What is your pre-tax, annual household income level? *Please check one.*

| □ less than $10,000 | □ $60,00 to $69,999 |  |
| --- | --- | --- |
| □ $10,00 to $19,999 | □ $70,00 to $79,999 |  |
| □ $20,00 to $29,999 | □ $80,00 to $89,999 |  |
| □ $30,00 to $39,999 | □ $90,00 to $99,999 |  |
| □ $40,00 to $49,999 | □ $100,00 or more |  |
| □ $50,00 to $59,999 |  |  |

*IF YOU ARE A COLLEGE STUDENT, PLEASE ANSWER THE FOLLOWING QUESTION.*

| **Only for respondents who are college students** | (F.8) Which class best describes your status as a college student? *Check one.*   \| □ Freshman \| □ Sophomore \| □ Junior \| \| --- \| --- \| --- \| \| □ Senior \| □ Graduate student \| □ Other \| |
| --- | --- | --- | --- | --- | --- | --- | --- |

*IF YOU ARE NOT A COLLEGE STUDENT, PLEASE ANSWER THE NEXT TWO QUESTIONS.*

| **Only for respondents who are not college students** | (F.9) Are you the primary shopper for your household? *Please check one.*   \| □ Yes \| \| --- \| \| □ No \| \| □ I share equally in the food purchasing decisions \| |
| --- | --- | --- | --- | --- |
|  | (F.10) What is your relationship with OSU? *Please check one.*   \| □ Faculty \| \| --- \| \| □ Staff \| \| □ Other employment by OSU \| \| □ I am not employed by OSU \| |

(F.11) Overall, what did you think of your experience today? *Please check all that apply.*

| □ I liked the food | □ I liked the atmosphere |
| --- | --- |
| □ The taste test was fun | □ The directions were clear and easy to follow |
