# Supplementary material for: Preference evaluation of ground beef by untrained subjects with three levels of finely textured beef
Source: PLoS One. 2018 Jan 17;13(1):e0190680. doi: 10.1371/journal.pone.0190680 (PMC5771606; doi:10.1371/journal.pone.0190680)
Supplement: S1 Table — (DOCX) [file pone.0190680.s002.docx]

**S1 Table.** **Using the Benjamin-Hochberg method to correct for multiple testing (all 27 tests)**

|  |  |  |  | Benjamin-Hochberg Critical Values | |
| --- | --- | --- | --- | --- | --- |
| Sensory Evaluation | **Type of Statistical Test** | **Description of Null Hypothesis** | **P-Value of Null** | **5% False Discovery Rate** | **10% False Discovery Rate** |
|  |  |  |  |  |  |
| 1st sensory evaluation: juiciness of plain beef | parametric simulations | same latent juiciness score for no FTB and 15% FTB | 0.0057 | 0.0019 | **0.0037** |
| 1st sensory evaluation: tenderness of plain beef | parametric simulations | same latent tenderness score for 15 FTB and >15% FTB | 0.0078 | 0.0037 | **0.0074** |
| 1st sensory evaluation: tenderness of plain beef | parametric simulations | same latent tenderness score for no FTB and 15% FTB | 0.0092 | 0.0056 | **0.0111** |
| 1st sensory evaluation: tenderness of plain beef | likelihood-ratio test | coefficients in ordinal-logit same for all beef types | 0.0099 | 0.0074 | **0.0148** |
| 1st sensory evaluation: juiciness of plain beef | likelihood-ratio test | coefficients in ordinal-logit same for all beef types | 0.0209 | 0.0093 | 0.0185 |
| 1st sensory evaluation: overall satisfaction of plain beef | parametric simulations | same latent overall satisfaction score for 15 FTB and >15% FTB | 0.0259 | 0.0111 | 0.0222 |
| 2nd sensory evaluation: overall satisfaction of slider | parametric simulations | same latent overall satisfaction for no FTB and >15% FTB (after 1st bite) | 0.0500 | 0.0130 | 0.0259 |
| 1st sensory evaluation: flavor of plain beef | parametric simulations | same latent flavor score for 15 FTB and >15% FTB | 0.0577 | 0.0148 | 0.0296 |
| 1st sensory evaluation: overall satisfaction of plain beef | likelihood-ratio test | coefficients in ordinal-logit same for all beef types | 0.0729 | 0.0167 | 0.0333 |
| 1st sensory evaluation: flavor of plain beef | parametric simulations | same latent flavor score for no FTB and >15% FTB | 0.0846 | 0.0185 | 0.0370 |
| 1st sensory evaluation: flavor of plain beef | likelihood-ratio test | coefficients in ordinal-logit same for all beef types | 0.1105 | 0.0204 | 0.0407 |
| 1st sensory evaluation: overall satisfaction of plain beef | parametric simulations | same latent overall satisfaction score for no FTB and 15% FTB | 0.1105 | 0.0222 | 0.0444 |
| 2nd sensory evaluation: overall satisfaction of slider | parametric simulations | same latent overall satisfaction score for 15% FTB after 1st bite and when finished eating | 0.12 | 0.0241 | 0.0481 |
| 1st sensory evaluation: juiciness of plain beef | parametric simulations | same latent juiciness score for no FTB and >15% FTB | 0.1614 | 0.0259 | 0.0519 |
| 2nd sensory evaluation: overall satisfaction of slider | parametric simulations | same latent overall satisfaction score for >15% FTB after 1st bite and when finished eating | 0.17 | 0.0278 | 0.0556 |
| 1st sensory evaluation: juiciness of plain beef | parametric simulations | same latent juiciness score for 15 FTB and >15% FTB | 0.1703 | 0.0296 | 0.0593 |
| 2nd sensory evaluation: overall satisfaction of slider | parametric simulations | same latent overall satisfaction score for no FTB and 15% FTB (after 1st bite) | 0.24 | 0.0315 | 0.0630 |
| 2nd sensory evaluation: overall satisfaction of slider | parametric simulations | same latent overall satisfaction score for 15 FTB and >15% FTB (after finished eating) | 0.31 | 0.0333 | 0.0667 |
| 2nd sensory evaluation: overall satisfaction of slider | parametric simulations | same latent overall satisfaction score for no FTB and 15% FTB (after finished eating) | 0.34 | 0.0352 | 0.0704 |
| 2nd sensory evaluation: overall satisfaction of slider | parametric simulations | same latent overall satisfaction score for 15 FTB and >15% FTB (after 1st bite) | 0.39 | 0.0370 | 0.0741 |
| 2nd sensory evaluation: overall satisfaction of slider | likelihood-ratio test | coefficients in ordinal-logit same for all beef types | 0.5383 | 0.0389 | 0.0778 |
| 1st sensory evaluation: overall satisfaction of plain beef | parametric simulations | same latent overall satisfaction for no FTB and >15% FTB | 0.5412 | 0.0407 | 0.0815 |
| 2nd sensory evaluation: overall satisfaction of slider | parametric simulations | same latent overall satisfaction score for no FTB after 1st bite and when finished eating | 0.56 | 0.0426 | 0.0852 |
| 3rd sensory evaluation: utility from being slider | likelihood-ratio test | coefficients in conditional logit same for all beef types (when sold at same price) | 0.6159 | 0.0444 | 0.0889 |
| 1st sensory evaluation: flavor of plain beef | parametric simulations | same latent flavor score for no FTB and 15% FTB | 0.8714 | 0.0463 | 0.0926 |
| 2nd sensory evaluation: overall satisfaction of slider | parametric simulations | same latent overall satisfaction for no FTB and >15% FTB (after finished eating) | 0.94 | 0.0481 | 0.0963 |
| 1st sensory evaluation: tenderness of plain beef | parametric simulations | same latent tenderness score for no FTB and >15% FTB | 0.9514 | 0.0500 | 0.1000 |
